# A Giant Malignant Solitary Fibrous Tumor of the Pancreas: A Case Report and Review of the Literature

**DOI:** 10.7759/cureus.61467

**Published:** 2024-05-31

**Authors:** Atl Simon Arias Rivera, Marianna Nesme Vara, Moises Brener Chaoul, Marco A De La Rosa Abaroa, Rafael Padilla Longoria

**Affiliations:** 1 General Surgery, Hospital Angeles Lomas, Huixquilucan, MEX; 2 Surgical Oncology, Hospital Angeles Lomas, Huixquilucan, MEX

**Keywords:** tumor, malignant, benign, mesenchymal tumors, pancreas

## Abstract

We present a case report of a giant solitary fibrous tumor (SFT) with a review of the literature and discuss its biological features and diagnosis.

A 43-year-old man presented to our emergency department with abdominal pain and distension with an evolution of two days. Contrast-enhanced computed tomography (CT) showed a large, well-circumscribed semisolid mass (12 cm x 10 cm x 12 cm) localized in the pancreatic head. The histological diagnosis obtained by endoscopic ultrasound-guided trans-duodenal tumor biopsy with fine-needle aspiration showed proliferating short spindle-shaped cells, suggesting a mesenchymal neoplasia of low grade. We proceeded to a Whipple surgical technique. The histopathological study of the resected tumor confirmed proliferating spindle-shaped cells in the tissue, and one mitotic figure was observed in 10 high-power fields (HPFs). Immunostaining was positive for CD34 and STAT-6. The histological diagnosis was a malignant pancreatic SFT. In the six months posterior to the surgical procedure, the patient has been free of recurrent disease.

Preoperative diagnosis is difficult and requires comprehensive evidence including clinical, immunohistochemistry, and histological features. Since there are currently no recognized best practices, we advise total surgical excision and careful clinical monitoring.

## Introduction

A solitary fibrous tumor (SFT) is an uncommon mesenchymal tumor that tends to have a slow-growing nature and can develop in various parts of the body but is commonly seen in the pleura. It was first described in 1931 by Klemperer et al. [1].

SFTs are considered historically a separate entity, soft tissue and pleural hemangiopericytomas (HPCs) are now classified as SFTs. HPCs fall under the diagnosis of SFTs because of the overlapping histologic features and shared fusion transcript involving NAB2 and STAT6 [2].

SFTs are histologically characterized by the presence of irregularly organized ovoid to spindle cells, keloidal-type stromal collagen, and thin-walled, staghorn-shaped blood vessels [3]. SFTs of the pancreas are an exceptionally uncommon condition, with just 39 documented instances since 1999. The initial case was reported by Lüttges et al. This tumor does not exhibit any gender disparity and is primarily observed in middle-aged patients [4].

Due to their uncommon occurrence, documented examples of pancreatic SFTs have shown benign histopathological characteristics [3]. We provide a documented instance of pancreatic SFT with malignant characteristics, as verified through histopathological examination and immunohistochemical analysis.

## Case presentation

**Figure 1:**
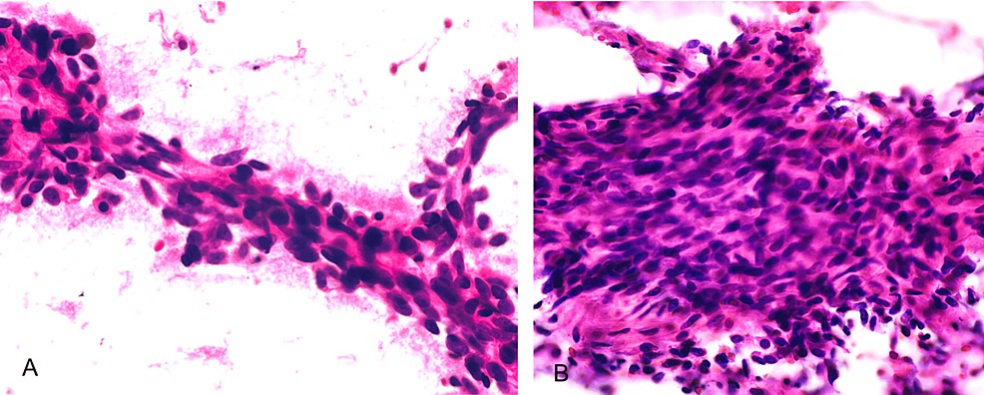
A and B: Biopsy of fine-needle aspiration (FNA). The cytological smears show low cellularity, fusiform and oval cells with wispy cytoplasm and pink collagenous stroma without atypia, mitosis, or pleomorphism.

A 43-year-old male, with a known history of mandibular sarcoma, was treated with surgical management with negative margins; there was no need for adjuvant therapy. The patient presented to our emergency department with abdominal distension, abdominal pain, and diarrhea with an evolution of two days. Laboratory studies did not show any alteration in liver function tests or tumor markers. Upon his arrival, we began the diagnostic process by conducting a computed tomography (CT) scan of the abdomen. The scan revealed an incidental, sizable, well-defined semisolid tumor measuring 12 cm x 10 cm x 12 cm located in the head of the pancreas. No abnormal accumulation indicating distant metastases or lymph node metastasis was seen. A trans-duodenal tumor biopsy was conducted using endoscopic ultrasound-guided fine needle aspiration (EUS-FNA). The histopathological study evidenced proliferating short spindle-shaped cells, suggesting a mesenchymal neoplasia of low grade. However, no definitive diagnosis could be given (Figure 1).

**Figure 2:**
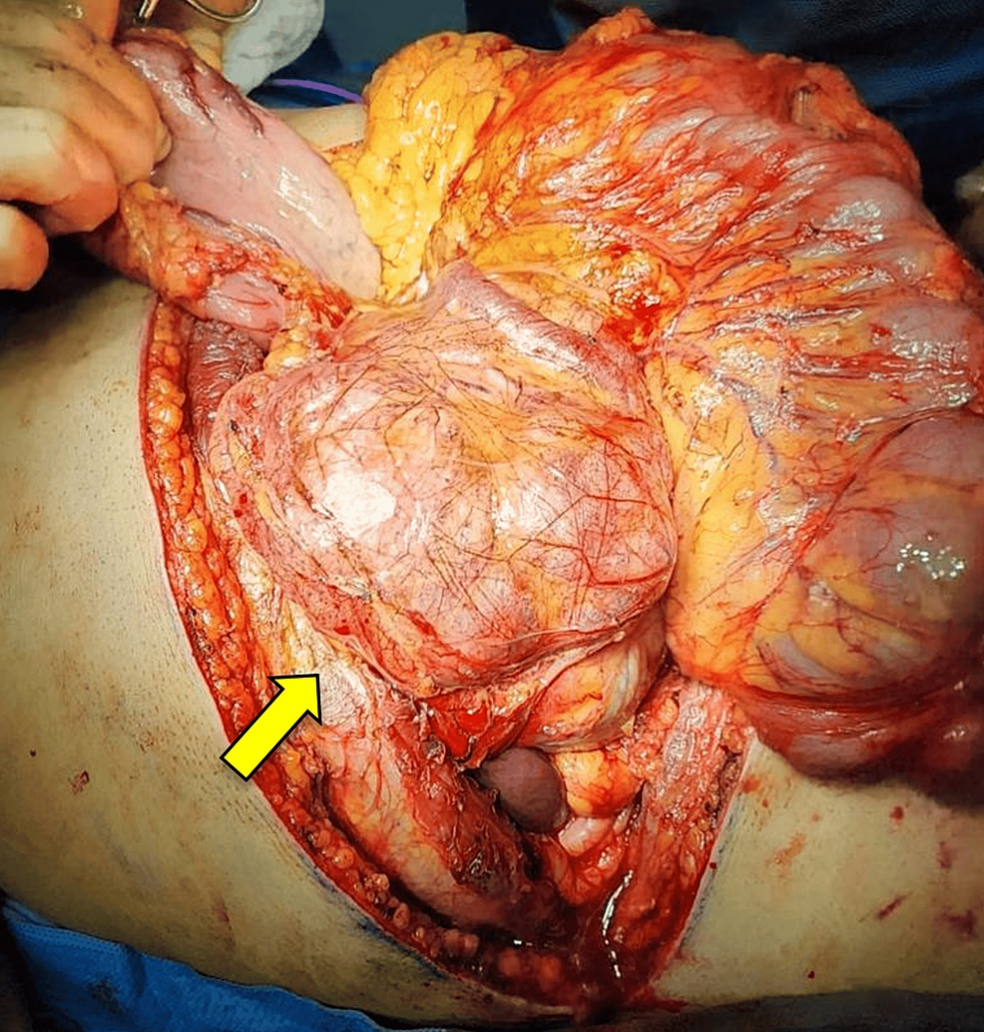
Exposure of the solitary fibrous tumor dependent on the pancreas head (yellow arrow).

**Figure 3:**
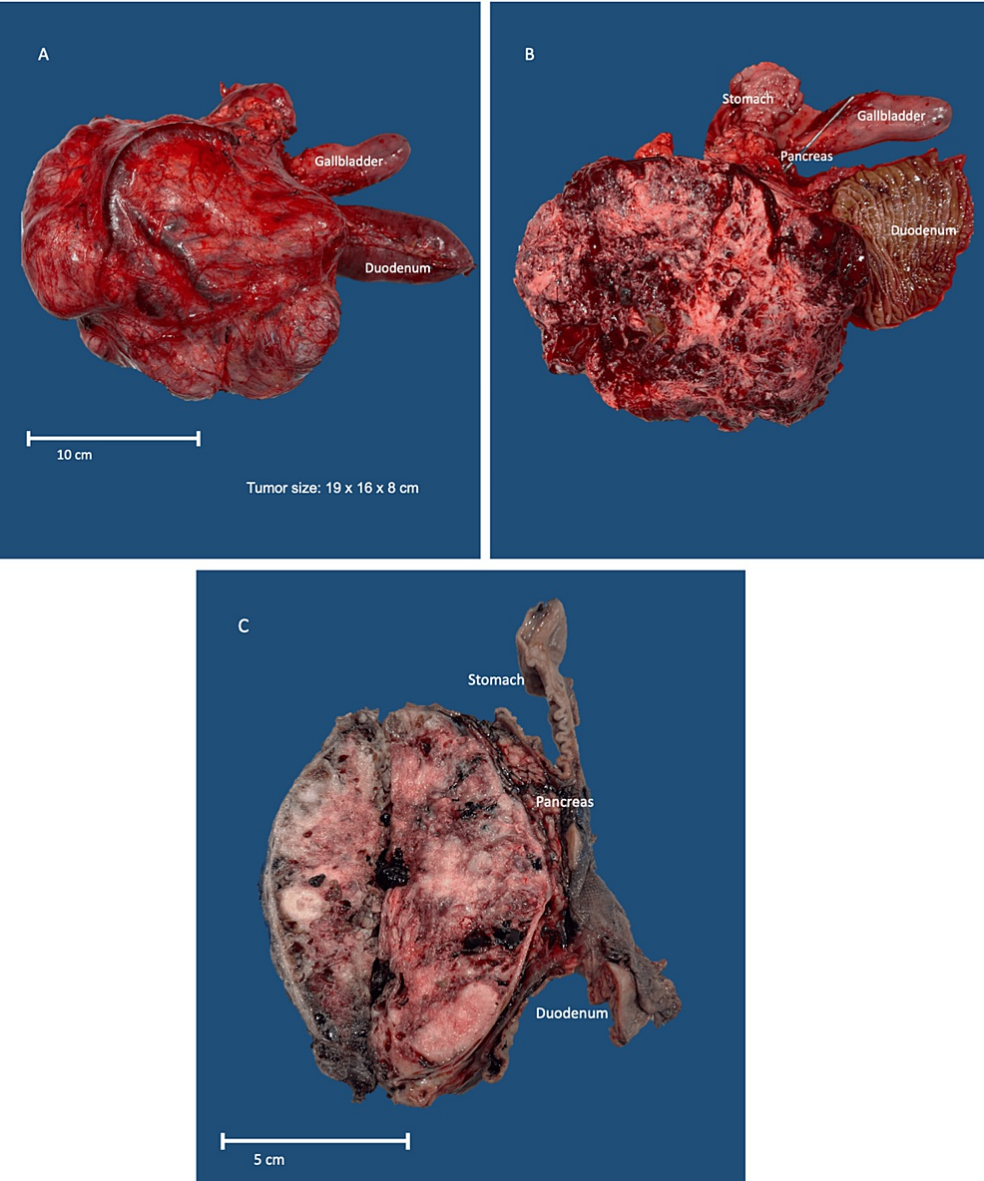
The tumor is lobulated with prominent vessels, compressing the pancreas and segment of the duodenum, without infiltrating them (A). Hemorrhagic cut surface with cystic degeneration and solid areas (B). Sagittal section of the tumor. The tumor is encapsulated, compressing the pancreas, stomach, and duodenum, without invasion (C).

**Figure 4:**
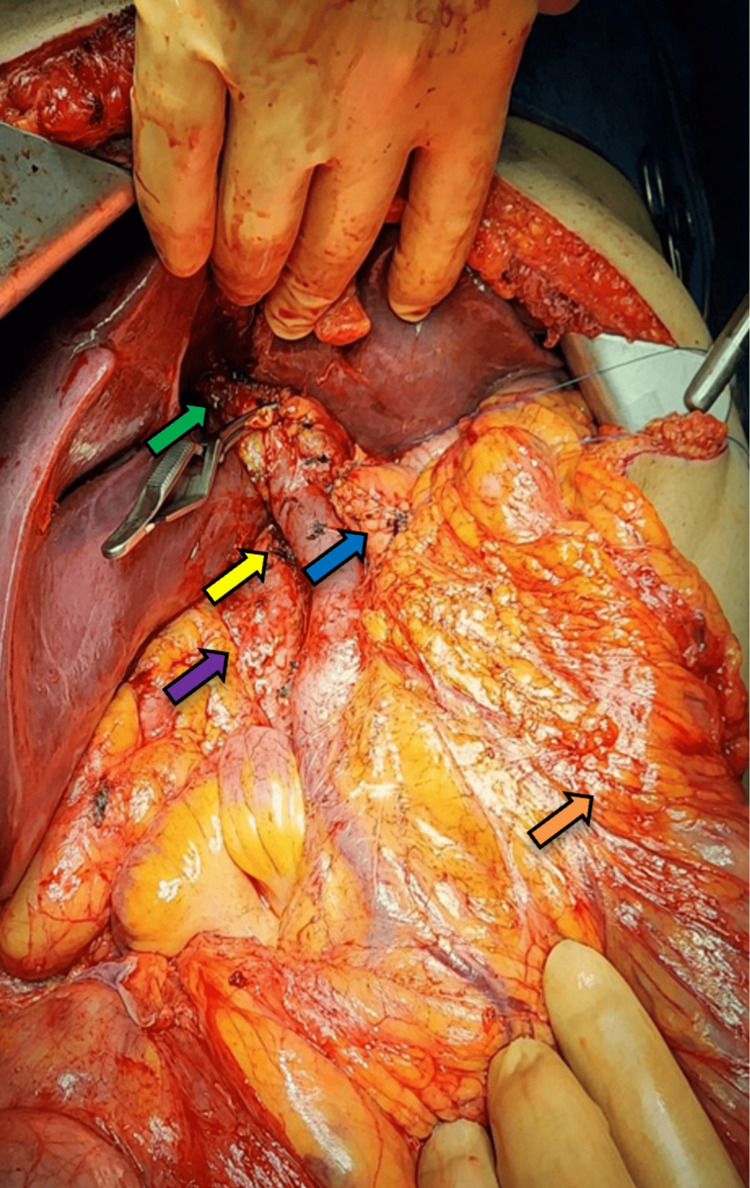
Excision of the tumor without lesion to major vessels: portal vein (yellow arrow), biliary tract (green arrow), pancreas (blue arrow), large bowel (orange arrow), and inferior vena cava (purple arrow)

A Whipple surgery was performed afterwards. The tumor was enclosed and measured 19 x 16 x 8 cm. It had a varied appearance, with a mixture of tan and white colors and a slightly heterogeneous pattern. There were also areas of hemorrhage within the tumor (Figure 2). The tumor was located at a distance from the edges of the proximal/gastric and distal/duodenal margins (Figure 3). The preservation of the inferior vena cava, superior mesenteric artery, and common hepatic artery was achieved by carefully separating them from the tumor (Figure 4).

**Figure 5:**
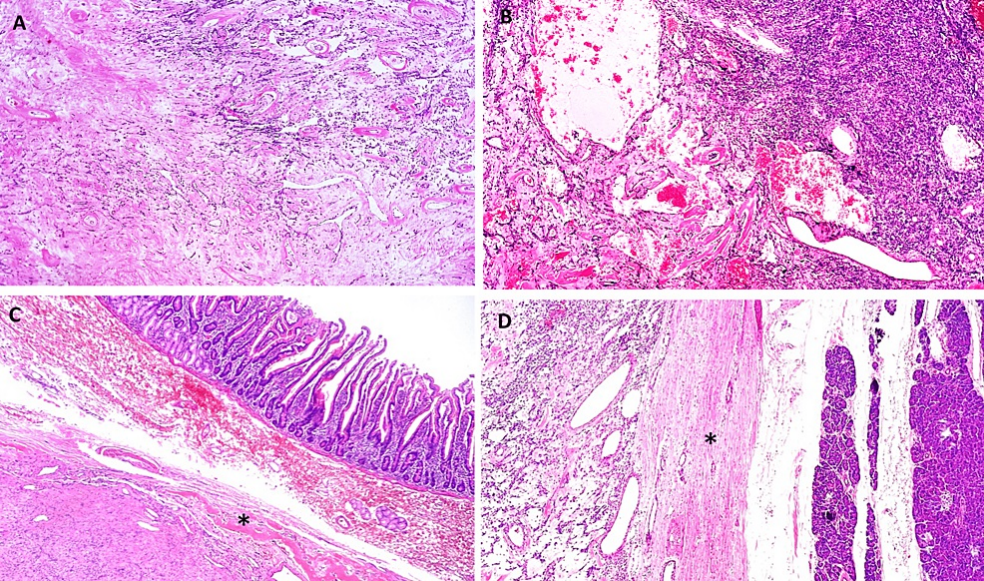
Microscopic features. Slides show a circumscribed spindled cell tumor with staghorn vasculature. A-B: Ovoid to fusiform spindle cells with indistinct cell borders arranged haphazardly and ill-defined fascicles. A: Hyalinized collagenous stroma and focally myxoid changes. B: Dilated, branching, hyalinized staghorn-like (hemangiopericytoma-like) vasculature. The tumor doesn´t infiltrate the duodenum (C) or the pancreas (D), it is surrounded by a sclerotic capsule (*).

Proliferating spindle-shaped cells involving normal pancreatic tissue were seen during pathological examination of the removed specimen. It was determined that most of the cells were excreting and that some were invasive (Figure 5).

**Figure 6:**
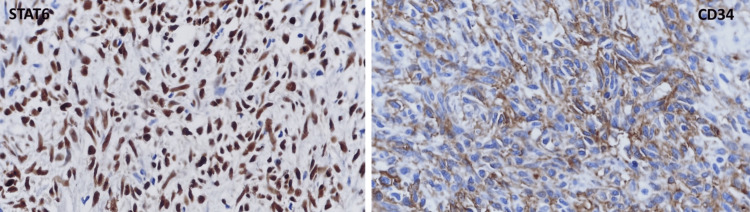
Immunohistochemical examination. This tumor was positive to STAT6 (nuclear) and CD34.

One mitotic figure was observed in 10 high-power fields (HPFs). The excised tumor's immunohistochemical examination showed that the tumor cells produced positive results for STAT-6 and CD34 (Figure 6). The tumor cells were negative for CD117, DOG-1, s-100 protein, SOX-10, and CKAE1/AE3. The tumor was identified as a malignant SFT of the pancreas based on the histology and immunostaining characteristics.

The patient's postoperative course was without complications. He was discharged seven days after the surgery disease free. A three-month follow-up was done at external consultation with no recurrence of disease.

## Discussion

This report provides a detailed description of the clinical and cytologic characteristics of a rare instance of SFTs affecting the pancreas.

 The SFT, as reported by Gold et al., is a type of tumor that makes up fewer than 2% of all soft tissue tumors [5]. It typically occurs in the thoracic cavity, but can also be found in various soft tissues and organs in the body [6].

Clinically the patients are either asymptomatic or present with non-speciﬁc ﬁndings such as abdominal pain or back pain, it is rare for the patients to present jaundice. SFTs of the pancreas are usually located in the head/neck with a median size of 4 cm. They are frequently diagnosed as pancreatic well-differentiated neuroendocrine tumors because of their well-circumscribed nature [7].

We conclude that the onset of presentation occurred incidentally in more than half of the literature reviewed, and we observed only seven case reports of abdominal pain as the first symptom to date.

SFTs are commonly non-cancerous and have a low likelihood of spreading to other parts of the body. Nevertheless, they have the tendency to exhibit aggressive behavior and possess a high likelihood of becoming malignant [8].

Originating from the pancreas, the SFT is an exceptionally rare condition, with just 39 recorded instances since 1999. The first case was described by Lüttges et al. [4].

A systematic review of the literature was carried out in the PubMed database with a result of 38 cases of SFTs dependent on the pancreas.

In the CT, the pancreatic SFT presents as a well-circumscribed tumor with an internal varied contrast effect, and in the MRI, it shows a hypointense on T1WI and hyperintensity on T2WI. The diagnosis is difficult in this tumor because its features are atypical [9].

The tumor was located in the head of the pancreas as a well-defined semisolid mass in our case, evidenced by the abdomen CT. There is a possibility of considering MRI as the next course of action; however due to the specific location of the tumor, we choose to proceed with EUS-FNA. Based on the information described earlier, we have determined that possible diagnosis for the tumor should include malignant lymphoma, neuroendocrine tumor, and SFT. Unfortunately, it was not possible to obtain a preoperative diagnosis. The histopathological investigation revealed the presence of rapidly dividing, elongated cells, indicating a low-grade mesenchymal tumor. Nevertheless, due to the lack of a conclusive diagnosis, we opted for surgery as the chosen course of treatment.

The SFT exhibits two histological characteristics: a patternless morphology characterized by the random growth of elliptical spindle-shaped tumor cells and a hemangiopericytic growth pattern resulting from vascular proliferation and perivascular sclerosis. The main microscopic differential diagnoses include desmoid tumor/ ﬁbromatosis, inﬂammatory myoﬁbroblastic tumor, schwannoma, GIST, and leiomyoma. Also, melanoma should always be considered in the differential diagnoses of spindle cell lesions [7].

Because SFTs fall within the classification of soft tissue tumors, these are classified with the TNM scale. In our case, this SFT of the pancreas was grade 1 [10].

Additionally, it is imperative to conduct an evaluation to determine the likelihood of malignancy. This assessment is contingent upon various factors including tumor size (exceeding 10 cm), mitotic activity (exceeding four mitotic figures per 10 HPFs), the existence of necrosis or bleeding, cellularity, nuclear pleomorphism, and/or vascular invasion. SFTs are typically identified by the presence of STAT6, Bcl 2, CD99, and CD34 proteins when examined using immunohistochemistry. Typically, they lack expression of epithelial membrane antigen (EMA) and S-100 [11].

SFTs are characterized by the fusion of nerve growth factor-induced (NGFI)-A binding protein 2 (NAB2) with signal transduction and activator of transcription 6 (STAT6), which leads to the overexpression of STAT6 in the cell nucleus as detected by immunohistochemistry [11].

Comparing these malignant criteria with our case, the patient had all the malignant factors except the mitotic figure, which was 1 mitotic figure in the tumor of the patient.

Based on our review of the literature, there has only been six cases of pancreatic SFTs with malignant findings, also 21 of the 38 cases currently reported performed a pancreatic resection to obtain free margins of the organs adjacent to the lesion.

The periodic follow-up with image examination is recommended in patients with malignant SFTs because of the probability for recurrence and metastasis. This is why the follow up in our patient has to be constant [10].

Currently, there is no documented therapy or treatment given after surgery or in case of recurrence. The preoperative radiation therapy and chemotherapy regimens have not shown overall efficacy, and there are no established standardized therapies [6].

However, similar to other soft tissue tumors, surgical intervention is the primary approach for treating SFTs, with a focus on achieving complete removal of the tumor. Research has demonstrated that achieving sufficient negative margins can reduce the likelihood of local disease recurrence and enhance survival rates [10,11].

According to the information provided, we conclude that radical surgical resection is still considered the most effective treatment option [12].

There have been a number of studies with postoperative radiotherapy especially in cerebral SFTs where the results showed that postoperative radiotherapy (PORT) intensity-modulated radiotherapy was helpful to prolong disease-free survival in patients compared with PORT stereotactic radiosurgery. Gou et al. showed that surgical resection and PORT were both superior to other types of treatment strategies [12]. We think that this type of strategy could be implemented or considered with irresectable or malignant tumors in the pancreas. A summary of histological features and outcomes of pancreatic SFTs is shown in Table 1.

**Table 1:** Histological features and outcomes of pancreatic solitary fibrous tumors. HPF: High-power field; SPF: solitary fibrous tumor

Author	Positive Inmunohistochemistry	Malignant features	Diagnosis of malignant SFT	Recurrence	Outcomes	Follow-up
Lüttges et al. 1999 [4]	CD34, CD99, Bcl-2, vimentin	No	No	No	Alive	20 mo
Chatti et al. 2006 [13]	CD34, CD99, Bcl-2, vimentin	No	No	No	Died postoperative complications	3 d
Gardini et al. 2007 [14]	CD34, CD99, Bcl-2, vimentin, SMA (focal)	NA	No	No	Alive	16 mo
Miyamoto et al. 2007 [15]	CD34, Bcl-2	No	No	No	Alive	7 mo
Srinivasan et al. 2008 [16]	CD34, Bcl-2	No	No	No	Alive	7 mo
Kwon et al. 2008 [17]	CD34, CD99, vimentin	No	No	No	NA	NA
Ishiwatari et al. 2009 [18]	CD34, Bcl-2	Necrosis	No	No	Alive	42 mo
Chetty et al. 2009 [19]	CD34, CD99, Bcl-2	No	No	No	Alive	6 mo
Sugawara et al. 2010 [20]	CD34	No	No	No	NA	NA
Santos et al. 2012 [21]	CD34, beta-catenin	No	No	No	NA	NA
Tasdemir et al. 2012 [22]	CD34, Bcl-2, beta-catenin, vimentin, Ki67 <2%	No	No	No	Alive	3 mo
Azadi et al. 2012 [9]	CD34, Bcl-2, Ki67 <5%	No	No	No	NA	NA
Van der Vorst et al. 2012 [23]	CD34, CD99, Bcl-2	No	No	No	NA	NA
Yamanashi et al. 2012 [24]	CD34, vimentin, Bcl-2	Intra-pancreatic metástasis, necrosis, >2 mitoses/HPFs, hypercellularity	Yes	Intra-pancreatic	Alive	32 mo
Chen et al. 2013 [25]	CD34, Bcl-2, vimentin, cd68, muscle-specific actin	Necrosis	No	No	Alive	30 mo
Hwang et al. 2014 [26]	CD34,Bcl-2, muscle-specific actin, CD10, ER, PR	No	No	No	Alive	30 mo
Han et al. 2015 [27]	CD34, CD99	No	No	-	No progression	10 mo
Estrella et al. 2015 [28]	CD34, Bcl-2, keratin (rare), p16, p53	Nuclear atypia, necrosis 17 mitoses/10HPFs	Yes	No	Alive	40 mo
Baxter et al. 2015 [29]	CD34, Bcl-2	NA	No	No	NA	NA
Paramythiotis et al. 2016 [30]	CD34, CD99, Bcl-2, vimentin, S-100 (focal)	No	No	No	Alive	40 mo
Murakami et al. 2016 [31]	STAT6, CD34, Bcl-2, ACTH (focal), POMC (focal), NSE (focal)	No	No	No	Died sepsis	4 mo
Spasevska et al. 2016 [32]	CD34, vimentin, CD99, Bcl-2 (focal), nuclear beta-catenin (focal)	No	No	No	Died postoperative complications	1 wk
Clare et al. 2017 [33]	STAT6, CD34, Bcl-2, CD56, cytokeratin CAM5.2, AE1/AE3	6/10 HPFs	Yes	No	Alive	40 mo
Sheng et al. 2017 [34]	CD34, vimentin, sma (focal), Ki67 <3%	Mild-moderate nuclear pleomorphism 2-5/10 HPFs hypercellularity	No	No	Alive	12 mo
D´Amico et al. 2017 [35]	STAT6, CD34	No	No	No	Alive	24 mo
Oana et al. 2017 [36]	CD34, Bcl-2	No	No	No	Alive	36 mo
Geng et al. 2020 [37]	STAT6, CD34, Bcl-2, CD31, PHH-3, D2-40, Ki67 >10%	4-5/10 HPFs necrosis	Yes	Residual liver tumor (+)	Alive	6 mo
Qian et al. 2020 [38]	STAT6, CD34, Bcl-2, Ki67 10%	Heterotypic cell 4-5/10 HPFs local infarction	Yes	No	Alive	10 mo
Taguchi et al. 2020 [39]	STAT6, CD34, Bcl-2, vimentin, cytokeratin AE1/AE3 (focal)	Hypercellularity 12/10 HPFs necrosis invasive growth	Yes	No	Alive	12 mo
Present case 2023	CD34, STAT6, Ki67 1%	Necrosis <10%, mitoses 1/10	Yes	No	Alive	3 mo

In this review of the literature, we provide an update of the new cases that have been published since the year 1990. Carrying out an exhaustive review of the literature, we found that only Tasdemir et al. in 2012 reported similar dimensions of the lesion found in our patient, thus considering it an exceptional presentation [22]. 

## Conclusions

Ultimately, it is uncommon for mesenchymal tumors to affect the pancreas, as seen in our example. Due to the various ways in which they are presented, diagnosing these conditions can be difficult as their radiological findings may lack specificity. A definitive diagnosis requires histopathological investigation and thorough ancillary testing. Due to the distinct malignant characteristics, it is advisable to conduct thorough monitoring and evaluation.
